# Case Report: Stage-by-stage fueling, glucose dynamics, and next-day metabolism and biomarker responses after baseline testing in an 18.5-hour Swedish classic tetrathlon

**DOI:** 10.3389/fspor.2026.1733702

**Published:** 2026-03-05

**Authors:** Jonny Trinh, Fredrik Edin, Ulrika Andersson-Hall, Stefan Pettersson

**Affiliations:** 1 Center for Health and Performance, Department of Food and Nutrition, and Sport Science, University of Gothenburg, Gothenburg, Sweden; 2 Institute of Neuroscience and Physiology, Department of Physiology, Sahlgrenska Academy, University of Gothenburg, Gothenburg, Sweden

**Keywords:** carbohydrate intake, case report, continuous glucose monitoring, oral glucose tolerance test, ultra-endurance

## Abstract

**Background:**

Amateur ultra-endurance (UE) athletes often exhibit suboptimal fueling, particularly inadequate carbohydrate (CHO) intake, during competition. Integrated real-world datasets that combine weighed-back, stage-specific fueling with blinded continuous glucose monitoring (CGM) and next-day oral glucose tolerance test (OGTT) including indirect calorimetry in a condensed multi-discipline UE setting are scarce.

**Case presentation:**

A 37-year-old amateur athlete completed a tightly timed “Swedish Classic” on official race courses within 18:30 h:min (18.5 h)—316 km road cycling, 3 km open-water swimming, 84 km roller-skiing, and 30 km trail running—with helicopter transfers between stages [433 km total; 15:01 h:min (15.0 h) active exercise]. Fasting laboratory tests were performed at 07:30 on the mornings before and after the attempt, including venous blood sampling, dual-energy x-ray absorptiometry (DXA), and a 75-g oral glucose tolerance test (OGTT; 120 min) with indirect calorimetry. Weighed-back nutrition and hydration, CGM, heart rate (HR), body mass (BM), and stage logistics were recorded throughout.

**Results:**

Total energy intake was 5,825 kcal with 1,051 g CHO (13.8 g·kg^−1^), averaging 57 g·h^−1^ including transfers and 50 g·h^−1^ during exercise. BM decreased by 2.5 kg (−3.8%). CGM showed stable glucose during cycling, swimming, and roller-skiing, with transient hypoglycaemia confined to the final run. Mean relative HR across all events was 72 ± 6%, and gastrointestinal symptoms were minimal. Next-day OGTT total glucose AUC was unchanged but showed a higher early peak and earlier nadir. Indirect calorimetry indicated reduced CHO oxidation (−28%) and increased fat oxidation (+47%). Inflammatory and muscle-damage markers increased, while cardiac troponin I remained within reference limits.

**Conclusion:**

In this condensed UE tetrathlon case, discipline-specific feeding opportunities, rather than generic hourly targets, largely determined achievable CHO delivery, and glycaemic excursions occurred only during the terminal running stage. This integrated case provides a reference observation to inform logistics-aware fueling plans and to motivate prospective studies evaluating whether real-time CGM with predefined decision rules improves late-stage fueling and glycaemic stability in UE events.

## 1 Introduction

Ultra-endurance (UE) events—often defined as exercise exceeding 6 h—impose substantial metabolic and gastrointestinal stress (1, 2). Suboptimal fueling remains common among amateur participants despite contemporary guidance to target 60–90 g·h^−1^ of carbohydrate (CHO), particularly via multiple-transportable CHO during prolonged exercise (3, 4). Yet integrative, real-time descriptions linking planned vs. achieved intake with continuous glucose monitoring (CGM), body mass (BM) changes, heart-rate (HR) load, and post-event metabolic testing are scarce (5). Moreover, while exercise training generally improves insulin sensitivity, prolonged efforts can produce heterogeneous next-day glucose tolerance, underscoring the need to contextualize post-event oral glucose tolerance test (OGTT) results within actual fueling and workload (5, 6).

Here we present a single-case report of a 37-year-old amateur athlete who completed the official courses of the Swedish Classic tetrathlon (7) —cycling, open-water swimming, roller-skiing, and trail running—within 18:30 h:min (18.5 h), enabled by helicopter transfers. This is among the first reports to integrate weighed-back, stage-specific fueling with blinded CGM during a condensed multi-discipline UE attempt, together with next-day OGTT and indirect calorimetry. By synchronizing these measures with HR load, BM fluctuations, and blood biomarkers, the case provides a reference description of discipline-specific fueling feasibility and early recovery metabolic responses.

## 2 Case description

### 2.1 Participant and setting

The participant, a 37-year-old male amateur endurance athlete (177 cm, 76.3 kg, 10.1% body fat, VO₂max 66 mL·kg^−1^·min^−1^), reported an average weekly endurance training volume of 13.5 h during the year preceding the tetrathlon (June 17, 2021–June 17, 2022). The participant provided written informed consent for publication of de-identified data. No prior diagnosis of cardiometabolic, gastrointestinal, or endocrine disease was reported. The participant undertook the attempt as a planned performance challenge and did not report any presenting medical complaint or symptoms.

### 2.2 Diagnostic assessment, follow-up and outcomes

Fasted laboratory assessments were conducted at 07:30 on the mornings before and after the event, including venous blood sampling (Table 2) and body composition analysis by dual-energy x-ray absorptiometry (DXA; GE Lunar iDXA, GE Healthcare, Madison, WI, USA). The post-event assessment time was selected for logistical reasons and to standardize sampling under comparable overnight-fasted, morning conditions, at the same time of day and using the same procedures as the pre-event assessment; because the event finished at 06:02, this schedule resulted in testing at ∼26 h post-event and was intended to capture an early recovery “snapshot” rather than acute (within-hours) responses. A 75-g OGTT was performed pre- and post-event with capillary glucose samples collected every 15 min over 120 min. Postprandial substrate oxidation (CHO and fat) was assessed using indirect calorimetry. The corresponding stoichiometric equations used to calculate oxidation rates, along with detailed analytical procedures, are provided in Supplementary Methods 1.3.

### 2.3 Event logistics and timeline

The tetrathlon attempt commenced at 11:32 on 18 June 2022 and ended at 06:02 (Figure 2A), corresponding to a total elapsed time of 18.5 h. For stage-specific analyses, Table 1 reports the summed duration of timed segments (four exercise stages and three helicopter transfers; 18:19 h:min). The ∼11 min difference represents non-timed transition/handling time around stage endpoints (e.g., weighing, equipment handling, and waiting for helicopter departure), which was not assigned to any single stage or transfer.

**Table 1:** Summary of nutritional intake, energy balance, fluid balance and body-mass fluctuations during the 18.5-h tetrathlon.

				Event				
Variable	Cycling	Helicopter 1	Open water swimming	Helicopter 2	Roller-skiing	Helicopter 3	Running	Total
Race Data								
Race distance (km)	316	-	3	-	84	-	30	433
Time to complete (h:min)	08:07	01:20	00:41	00:37	03:28	01:21	02:45	18:19^#^
Nutritional Data								
EI (kcal)	2,209	1,146	-	401	640	1,197	232	5,825
CHO intake (g·kg^−1^)	6.9	1.5	-	0.8	2.1	1.7	0.8	13.8
CHO intake (g; g·h^−1^)	528; 65	113	-	62	158; 46	132	58; 21	1,051; 57
Planned CHO intake (g; g·h^−1^)	711; 88	142	40; 40	142	337; 97	32	233; 85	1,636; 90
Protein (g)	3	34	-	6	-	29	-	71
Fat (g)	7	60	-	14	-	61	-	143
H_2_O-intake (mL; mL·h^−1^)	4,010; 494	2,354	-	515	1,200; 346	737	130; 47	8,946; 484
Planned H_2_O-intake (mL)	3,120	424	250	424	1,560	-	1,042	6,820
Sodium (mg; mg·h^−1^)	2,945; 363	1,935	0	175	1,000; 288	1307	160; 58	7522; 407
Caffeine (mg)	0	100	0	100	0	0	200	400
Energy Expenditure and Balance								
EE (kcal; kcal·h^−1^)*	7851; 967	-	759; 1109	0	3744; 1034	0	2979; 1080	15,333
EE (kcal; kcal·h^−1^)	7831; 965	-	590; 862	0	2987; 825	0	2406; 872	13,814
EB (EI-EE)*	−5,642	0	−759	0	−3,104	0	−2,747	−9,509
EB (EI-EE)	−5,622	0	−590	0	−2,347	0	−2,174	−7,990
Body Mass and Effective Body Water Changes								
*Δ*body mass (g; %)	−1,000; −1.31	-	−100; −0.13	-	200; 0.26	-	−1,600; 2.09	−2,500; −3.79
*Δ*EBW	2,065	-	258	-	260	-	2,020	4,603
*Δ*EBWgly (1:1)	2,494	-	368	-	563	-	2,210	5,635
*Δ*EBW gly (1:3)	3,351	-	587	-	1,170	-	2,591	7,698

EE, energy expenditure; EB, energy balance; CHO, carbohydrate; EBW, effective body water; *Δ*EBWgly (1:1) and *Δ*EBWgly (1:3); EBW including water bound to glycogen at 1 g or 3 g H₂O per g glycogen. Asterisk (*) indicates calculated EE from stage-specific models; see Supplementary Methods. Total elapsed time was 18:30 (h:min). 18:19**^#^** sums timed stages and helicopter transfers; ∼11 min of unassigned transition time (e.g., equipment handling/brief waiting) is excluded.

**Table 2:** Pre- and post-tetrathlon blood markers.

Blood marker	Pre	Post	Reference interval	Change from baseline (%)
Cardiac troponin I (ng·L^−1^)	<5	6	<35	20
C-reactive protein (mg·L^−1^)	0.89	76	<3	8,439
Creatine kinase (µkat·L^−1^)	11	30	0.80–6.7	173
Testosterone (nmol·L^−1^)	7.94	7.46	8.3–30	−6
Cortisol (nmol·L^−1^)	311	414	102–535	33
T:C ratio	0.0,255	0.018	-	−29
Myoglobin (µg·L^−1^)	69	144	<90	109
Leucocytes (× 10^9^·L^−1^)	4.7	5.8	3.5–8.8	23

T:C, Testosterone to Cortisol.

**Figure 1:**
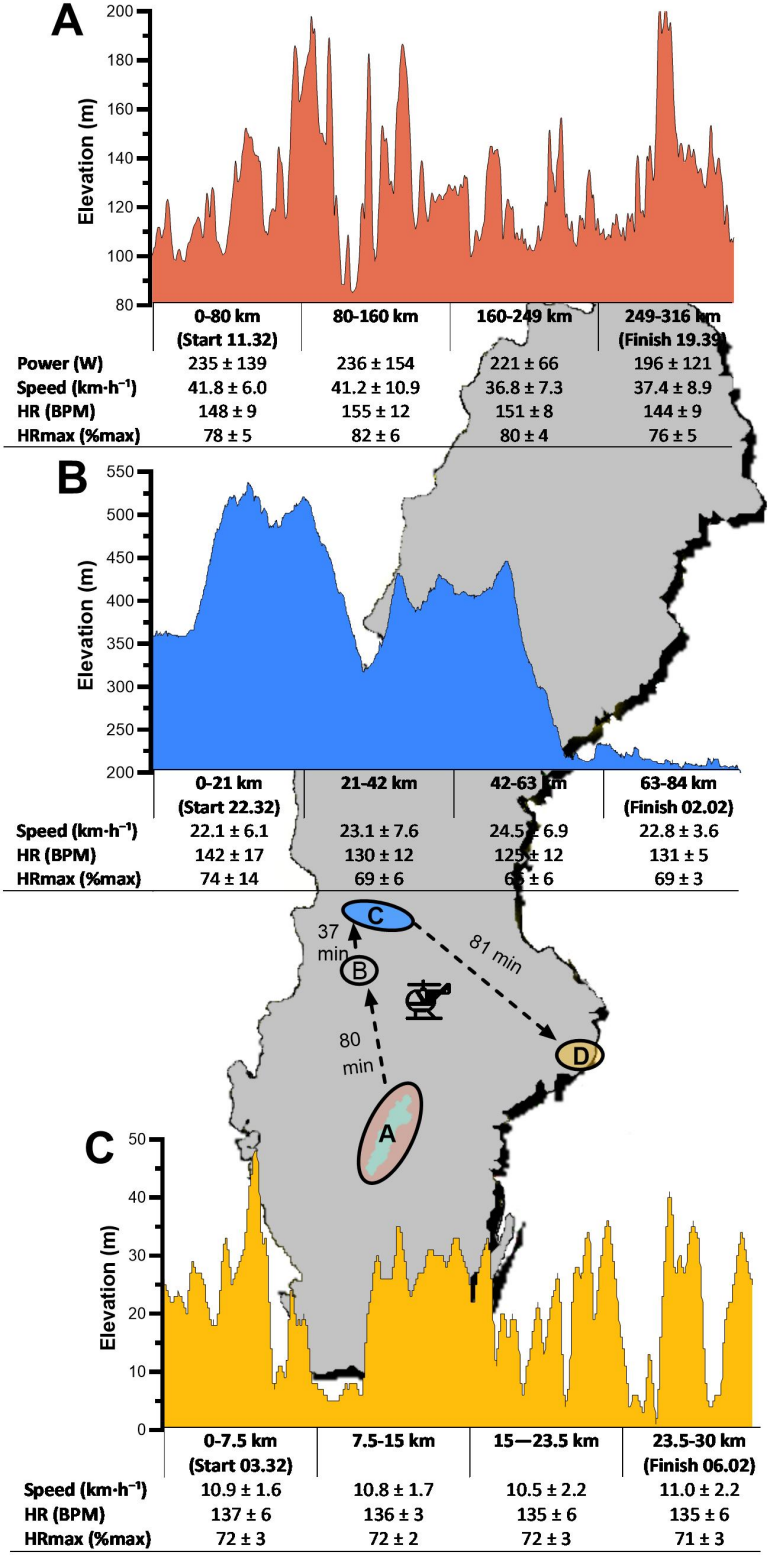
Course profiles, mean speed, heart rate (HR), and relative heart rate (%HRmax) across three events of the Swedish Classic completed within 18.5 h: **(A)** Vätternrundan (316 km cycling), **(B)** Vasaloppet (84 km roller-skiing), and **(C)** Lidingöloppet (30 km running). Helicopter transfers are indicated. Swim data (3 km, Vansbrosimningen) are not shown because elevation changes were negligible.

The attempt covered the official courses of Vätternrundan (316 km road cycling), Vansbrosimningen (3 km open-water swimming), Vasaloppet (84 km roller-skiing; off-season course), and Lidingöloppet (30 km trail running). Helicopter transfers enabled rapid transitions between venues. Vätternrundan was completed on the official event day (2022; 12216 participants), with a pre-arranged paceline of well-trained cyclists to enable cooperative drafting. The remaining stages were self-paced, with logistical/safety support from a support team and one member of the research team (JT). Course profiles are shown in Figure 1. Ambient temperature during the 18.5-h observation period ranged from 2 to 19.7 °C.

### 2.4 Nutritional plan vs. achieved intake

All nutrition and hydration were pre-planned with stage-specific hourly targets (Table 1). Meals, confectionery (e.g., chocolate bars) and sports products (drinks, gels bars) were prepared and weighed in advance and stored in insulated thermoses (Sausage Stroganoff with rice consumed during transfers). Sports products and confectionery were distributed at stage starts and official aid stations, and intended hourly intakes were later compared with achieved intakes using weighed-back methods and time-stamped logs. The plan specified 1,636 g CHO (target 90 g·h^−1^), approximately 6,800 mL fluid, and 10.4 g sodium across the monitored period.

### 2.5 Physiological monitoring

The athlete wore a Polar H10 chest strap paired with a Grit×Pro watch (Polar, Kempele, Finland; second-by-second HR sampling). Cycling energy expenditure was estimated from power using an individualized gross-efficiency model, while non-cycling energy expenditure was derived from fixed rates; full calculations are described in Supplementary Materials 1.1. Cycling metrics (time, speed, power, gradient, and elevation) were recorded using a GPS cycle computer (Element, Wahoo, Atlanta, GA, USA). Body mass (BM) was measured before and after each stage using a calibrated ±100 g scale. Effective body water (EBW) and sweat loss were estimated from BM change, recorded fluid intake, approximated urine output, and adjustments for metabolic water production and respiratory water loss (detailed in Supplementary Materials 1.2). Nutritional intake, including packaged products and prepared meals, was compiled from manufacturer labels and analyzed using Dietist NET software, version 2023.07.20 (Kost & Näringsdata, Sweden), which also enabled estimation of total fluid intake from both beverages and the water content of semi-solid and solid foods. Interstitial glucose was monitored with a factory-calibrated CGM (Freestyle Libre Pro IQ, Abbott Diabetes Care Inc, Alameda, USA), with 15-min logging. The sensor was applied during the pre-event laboratory assessments (day −1), and data collection was blinded (no real-time display/feedback to the participant).

### 2.6 Gastrointestinal assessment

Gastrointestinal (GI) symptoms (gas, bloating, abdominal discomfort, nausea, stomach rumbling, urgency to defecate, and abdominal pain) and overall digestive comfort (*extremely uncomfortable* to *extremely comfortable*) were rated immediately before and after each event on a 0–20 scale (0 = no symptoms, 20 = worst conceivable symptoms) (8).

## 3 Results

### 3.1 Performance outcomes

The athlete completed a total of 433 km in 18.5 h, including 15:01 h:min (15 h) of active exercise and 3:18 h:min (3.3 h) of helicopter transfers. With the exception of swimming, stage-specific completion times, speeds, and HR responses are summarized in Figure 1. During the 3-km open-water swim, mean swim speed remained stable (1.22 m·s^−1^), while mean %HRmax increased from 68 ± 6% to 78 ± 4% between the first and final kilometer. Mean relative HR across all events was 72 ± 6%.

### 3.2 In-race nutritional intake, energy balance and body mass

**Figure 2:**
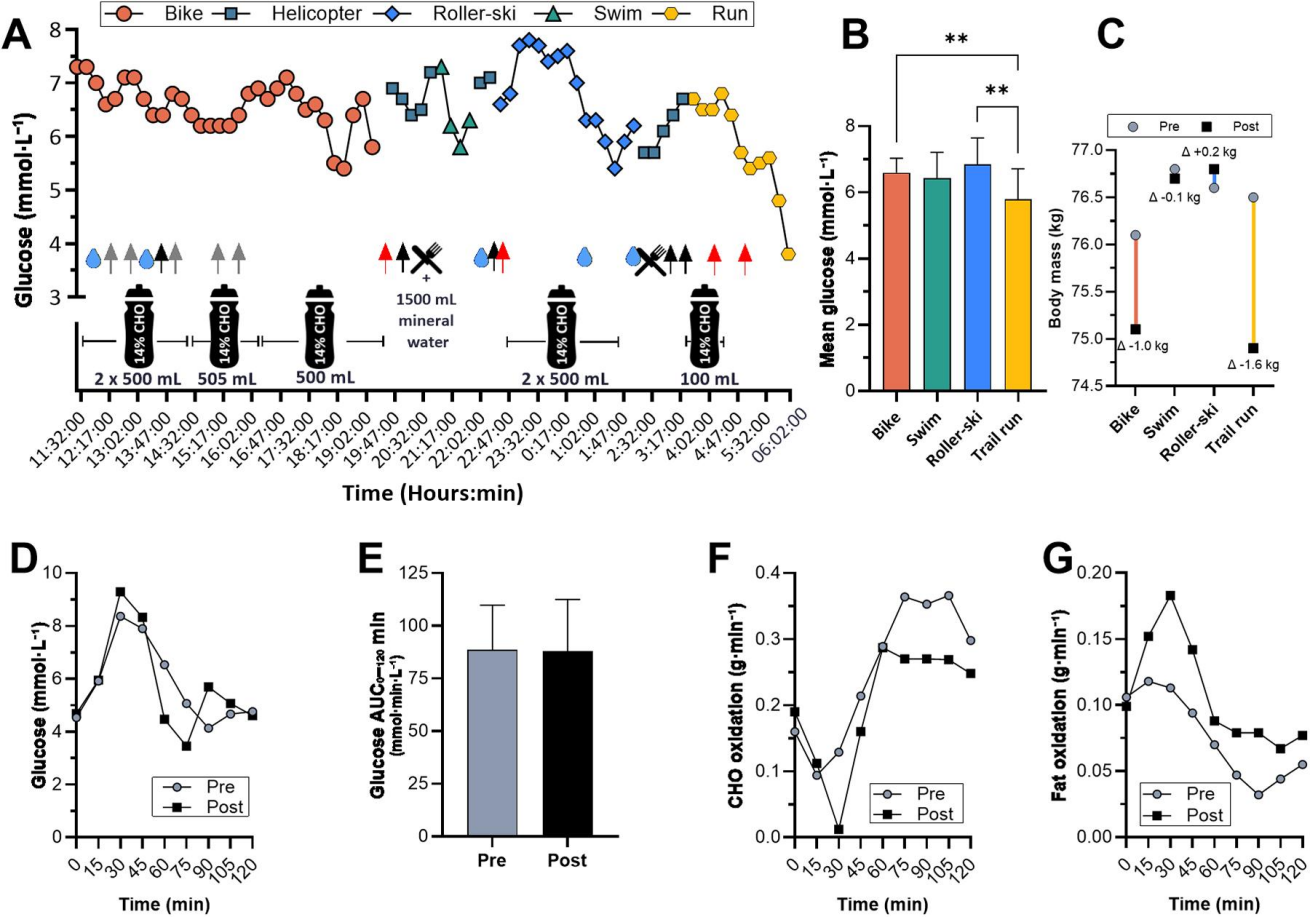
**(A)** interstitial glucose concentrations across events with concurrent nutritional intake, and **(B)** mean glucose per event. **(C)** Body mass before (Pre; grey circles) and after (Post; black squares) each stage. **(D)** Capillary glucose concentrations, **(E)** glucose AUC, and **(F,G)** carbohydrate and fat oxidation rates during a 75-g OGTT performed in the morning before (Pre; grey circles) and after (Post; black squares) the tetrathlon. ***Note.*** Grey, red and black arrows in **(A)** indicate intake of CHO-gel, caffeine–CHO-gel, chocolate bar, and 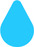
200-500 mL plain water, respectively. 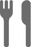 = solid food intake. Drink bottles indicate sports drink (14% CHO) consumption; the value below each bottle shows the amount consumed, and horizontal lines to the left and right of the bottle denote the time frame of intake. **p < 0.01 cycling vs. running and roller-skiing vs. running.

A consolidated 18.5-h timeline (Figure 2A) illustrates the sequence of stages, helicopter transfers, and key fueling episodes (drinks, gels, and solids). As shown in Table 1, total energy intake was 5,825 kcal corresponding to 38% of the estimated expenditure, (15,333 kcal). Total CHO intake was 1,051 g (13.8 g·kg^−1^), equating to an overall mean of 57 g·h^−1^ when helicopter transfers were included. When considering exercise time only, mean CHO intake was 50 g·h^−1^, with stage-specific rates of 65, 0, 46, and 21 g·h^−1^ during cycling, swimming, roller-skiing, and running, respectively. Sports drinks provided the largest share of ingested CHO (52%), followed by semi-solid gels (21%) and solid foods such as chocolate bars and cooked rice (26%). Fluid intake totaled 8,946 mL, approximately half of which was consumed during cycling. Total sodium intake was 7,522 mg, corresponding to 407 mg·h^−1^ overall and 273 mg·h^−1^ during active exercise.

Overall BM decreased by 2.5 kg (−3.8%), largely during running (−1.6 kg), despite positive EBW increase across events (Table 1 and Figure 2B). Relative to the nutrition plan, CHO intake adherence was ∼64%, fluid intake exceeded the target, and sodium intake was slightly below the recommended level. GI symptom scores were at the lower end (≤4) of the 20-point scale, with the highest recorded scores being 10 (= Neutral) for nausea post-cycling and 10 for gas/bloating pre–roller-ski (see Supplementary Figure S1).

### 3.3 In-event glucose dynamics

Despite a large energy deficit and sub-recommended average CHO delivery, interstitial glucose remained broadly stable during cycling, swimming, and roller-skiing, with hypoglycemic excursions (e.g., <3.9 mmol·L^−1^) confined to the final running stage (Figure 2).

### 3.4 Pre- and post-event blood markers

Pre–post changes in lean (−318 g) and fat mass (−32 g) were within the DXA's technical error and least significant change as later established for the same system in our laboratory (9). In contrast, biomarkers indicated substantial inflammatory and muscle-damage responses: C-reactive protein (CRP) increased from 0.89 to 76 mg·L^−1^; creatine kinase (CK) and myoglobin rose markedly; and the testosterone-to-cortisol ratio fell by ∼29%. Troponin I remained within reference limits (Table 2).

### 3.5 Pre- and post-event OGTT

OGTT profiles and metrics are shown in Figures 2D–G. Fasting and 120-min glucose values were similar pre- and post-tetrathlon, both within the normal range. Post-event, the glucose response showed a higher early peak (9.3 vs. 8.4 mmol·L^−1^) and an earlier nadir (60 vs. 75 min), while total AUC was unchanged. Substrate use shifted: mean CHO oxidation decreased by 28% and fat oxidation increased by 47% across the test. Metabolic flexibility, quantified as *Δ*RER₀–₆₀ (60-min minus baseline), was attenuated (+0.040 vs. +0.070), consistent with lower CHO stimulation and weaker fat suppression. Accordingly, *Δ*CHO₀–₆₀ was +0.129 vs. +0.097 g·min^−1^ (pre vs. post), and *Δ*FAT₀–₆₀ was −0.036 vs. −0.012 g·min^−1^, where negative values denote suppression relative to baseline.

## 4 Discussion

This case report provides a time-stamped description of planned vs. achieved nutrition and hydration, blinded interstitial glucose monitoring, physiological load, and next-day laboratory outcomes following completion of a tightly scheduled 18.5-h, four-discipline UE challenge. The primary contribution is descriptive: it documents how discipline-specific feeding opportunities aligned with glucose dynamics and how a next-day OGTT/indirect calorimetry assessment and biomarker panel appeared relative to baseline. Given the single-case design, blinded CGM, and a single post-event laboratory time point (∼26 h post), interpretations are necessarily hypothesis-generating.

### 4.1 Fueling relative to recommendations and practical determinants

Across the 18.5 h observation, the athlete ingested 1,051 g of CHO (13.8 g·kg^−1^), averaging 57 g·h^−1^ when helicopter transfers were included and approximately 50 g·h^−1^ during 15 h active exercise. This was below generic guidance for prolonged UE exercise (typically 60–90 g·h^−1^) (3, 4) and corresponded to 64% of the planned CHO target, warranting contextual framing.

The nutrition plan was developed independently by the participant and translated contemporary guideline targets into stage-specific allocations, without feasibility testing of modality-specific intake limits or rehearsal under race-equivalent conditions. While we cannot generalize from a single case, this type of guideline-derived planning may reflect circumstances where athletes lack access to specialized nutritional expertise. Foreseeable, discipline-specific feeding constraints were mirrored in the achieved CHO intake: ∼65 g·h^−1^ in cycling, 0 g·h^−1^ in open-water swimming, ∼46 g·h^−1^ in roller-skiing, and ∼21 g·h^−1^ during late-stage running. Accordingly, the case illustrates how guideline-based CHO targets may translate into lower achievable intakes under realistic, discipline-specific conditions when feasibility testing is not incorporated into planning, rather than representing an unanticipated deviation from an evidence-based strategy.

From a practical standpoint, fueling strategies may benefit from moving beyond uniform hourly targets across segments. Planning should incorporate modality-specific intake ceilings established during training under race-equivalent intensity and duration and front-load CHO delivery in segments where posture and hand availability favor high intake rates, with scheduled compensatory feeds during transitions or immediately following constrained segments. During late weight-bearing stages, low-viscosity, multiple-transportable CHO sources co-ingested with fluid may improve practical deliverability when voluntary intake declines (3, 10, 11). Prospective testing in amateur cohorts should verify whether such structured, modality-aware planning reliably elevates mean CHO delivery toward ≥60 g·h^−1^ without increasing gastrointestinal burden.

### 4.2 Hydration and sodium context

Total fluid intake was 8,946 mL and total sodium 7,522 mg across 18.5 h, alongside an overall BM change of −2.5 kg (−3.8%), with most of the loss occurring during the final run (−1.6 kg). Effective body water estimates were positive across stages (Table 1), although these estimates rely on assumptions regarding metabolic water production, respiratory losses, and approximated urine output. Collectively, the data indicate substantial fluid turnover during a temperate event (2–19.7 °C), but hydration status cannot be classified definitively because plasma osmolality/sodium and sweat sodium losses were not measured.

Fluid intake during the final run was very low (∼47 mL·h^−1^) and coincided with the lowest CHO intake rate and the period of declining interstitial glucose. Gastrointestinal function and processes relevant to CHO delivery (e.g., gastric emptying and intestinal absorption) were not assessed, which limits mechanistic interpretation in this case. Experimental studies indicate that beverage volume, CHO concentration, and exercise mode can influence gastric emptying and intestinal delivery (11, 12), but the present report uses the stage-specific intake pattern primarily as contextual information, while intervention-oriented recommendations require prospective testing.

### 4.3 Glycemic dynamics and exercise mode–specific factors

Interstitial glucose remained broadly stable during cycling, swimming, and roller-skiing, with hypoglycaemic excursions confined to the final running stage. This pattern is compatible with the pacing profile and the stage-specific intake distribution, particularly the higher CHO intake during cycling. Because CGM was blinded by design, glucose trends could not inform in-race decisions; thus, the present case is descriptive and does not test CGM-guided adjustment strategies.

From an evidence-weighted perspective, the most directly supported interpretation is that hypoglycaemia emerged during the terminal, weight-bearing stage in the context of markedly reduced CHO intake and accumulated energy deficit. Although the participant reported minimal GI symptoms, the dataset cannot determine whether late-stage glycaemic declines were influenced by altered CHO absorption, hepatic glucose output, or endocrine counter-regulation, because these processes were not directly measured. Field studies using CGM in UE settings report substantial inter-individual variability and correlations between CHO intake, glycaemic variability, and performance, but do not establish causality or isolate the effect of CGM visibility (13–16). Whether visible CGM combined with predefined decision rules can mitigate late-stage glycaemic decrements and/or improve performance in similar settings remains an open research question.

### 4.4 Post-event OGTT and blood markers

The next-day OGTT showed preserved total glucose AUC with a reshaped curve (higher early peak and earlier nadir), alongside lower CHO oxidation, higher fat oxidation, and reduced metabolic flexibility (*Δ*RER₀–₆₀). Biomarkers indicated a pronounced acute post-event response (elevated CRP, CK, and myoglobin; modest leukocytosis; reduced testosterone-to-cortisol ratio), while cardiac troponin I remained within the reference range.

Next-day reductions in glucose tolerance and insulin sensitivity have been reported after prolonged endurance exercise (5). In contrast, total glucose AUC in the present case was unchanged, indicating that next-day OGTT responses may vary across endurance and UE contexts. Direct comparison is limited by differences in exercise dose and modality, recovery timing, and post-event CHO restoration. Accordingly, the principal value of this case lies in transparent documentation of concurrent next-day metabolic responses and biomarker shifts at ∼26 h post-event rather than definitive physiological classification; serial follow-up is needed to establish time course, variability, and clinical relevance.

### 4.5 Strengths and limitations

This single-case, real-world report integrates time-stamped, weighed-back intake, blinded CGM, continuous HR monitoring, and paired laboratory testing surrounding a four-discipline challenge completed within 18.5 h. Limitations include the single-participant design, event-specific logistics (including helicopter transfers), estimated rather than directly measured fluid balance and non-cycling energy expenditure, interstitial CGM lag with 15-min logging, and laboratory follow-up limited to a single post-event time point (∼26 h). In addition, the nutrition plan was presented as stage-specific targets but was not documented as feasibility-tested across modalities, which limits interpretation of plan-vs.-achieved discrepancies. These constraints restrict causal inference, but do not detract from the dataset's value as a reference observation for future hypothesis-driven research.

## 5 Conclusions

This case illustrates how generic CHO intake targets may overestimate achievable delivery in condensed, multi-discipline UE settings when modality-specific feeding constraints are not explicitly feasibility-tested. The dataset provides a reference description linking stage-specific intake patterns with concurrent glycaemic dynamics and a next-day metabolic and biomarker snapshot. Future prospective studies should evaluate modality-aware fueling protocols under race-equivalent conditions and determine whether real-time CGM visibility combined with predefined decision rules improves late-stage fueling, glycaemic stability, and recovery trajectories in UE events.

### 5.1 Patient perspective

The tetrathlon experience taught him to treat the nutrition plan as non-negotiable and to prioritize disciplined adherence across all segments. During Vätternrundan on the official event day, he initially rode in a pre-arranged paceline but, after losing contact with the paceline, covered ∼100 km solo. He recognized that maintaining composure and following the pre-specified schedule should take precedence over tactical moves (e.g., trying to stay with the peloton) during cycling. The final, nighttime trail run exposed a gap between plan and appetite, as aversion to intake and lack of hunger curtailed feeding. In retrospect, he would have initiated gut-training with higher intakes much earlier, acknowledging the practical challenge of rehearsing nutrition under nocturnal conditions.

## Data Availability

The raw data supporting the conclusions of this article will be made available by the authors, without undue reservation.
