# Supplementary material for: Case Report: Stage-by-stage fueling, glucose dynamics, and next-day metabolism and biomarker responses after baseline testing in an 18.5-hour Swedish classic tetrathlon
Source: Front Sports Act Living. 2026 Mar 5;8:1733702. doi: 10.3389/fspor.2026.1733702 (PMC12999917; doi:10.3389/fspor.2026.1733702)
Supplement: Supplementary file 1 [file Datasheet1.docx]

Supplementary Material

Case report: Stage-by-stage fueling, glucose dynamics, and next-day metabolism and biomarker responses after baseline testing in an 18.5-hour Swedish Classic tetrathlon

Authors: Jonny Trinh, Fredrik Edin, Ulrika Andersson-Hall, Stefan Pettersson

**Contents**

[1 Supplementary Methods 1](#_Toc211255877)

[1.1 Metabolic energy expenditure calculations 1](#_Toc211255878)

[1.2 Estimation of Net Effective Body Water and Component Mass Changes 1](#_Toc211255879)

[1.3 Pre‑ and post‑event OGTT 4](#_Toc211255880)

[1.4 Gastrointestinal symptoms 4](#_Toc211255881)

[2 Supplementary Figures 5](#_Toc211255882)

[Supplementary Figure 1 5](#_Toc211255883)

[3 References 7](#_Toc211255884)

# Supplementary Methods

## Metabolic energy expenditure calculations

Metabolic energy expenditure (EE) during the cycling stage was estimated using Equation 1 together with the athlete’s individualized, power-specific gross efficiency (GE) derived from a prior graded cycling test, as described by Homestead et al. (2016). Fixed EE rates of 18.5 and 18.0 kcal·min⁻¹ were applied for the swim and trail run stages, respectively, based on data reported for a top-level amateur triathlete by Maunder et al. (2018). As a previous study found no significant differences in carbohydrate metabolism, EE, or metabolic equivalents (METs) between running and roller skiing during a 30-min submaximal trial, the same fixed EE rate as running was applied to roller skiing (Maunder et al., 2018).

Equation for estimating metabolic EE during cycling:

| $Metabolic EE \left( kcal \right)=\frac{Mechanical power \left( Watts \right)}{GE \left( fractional \right)} \times4.18$ | (1) |
| --- | --- |

Equation for estimating individualized power-specific GE (%):

| $y= a\times x^{4}+b\times x^{3}+c\times x^{2}+d\times x-e$ | (2) |
| --- | --- |

where y represents the individualized power-specific GE (%), x denotes mechanical power (Watts), and a, b, c, d, and e are constants derived from the polynomial regression model. The R² for the model was 0.99.

## Estimation of Net Effective Body Water and Component Mass Changes

This section summarizes the components and estimation procedures used to determine changes in effective body water (EBW). Three variants of EBW change are reported: one excluding glycogen-associated water (ΔEBW) and two including glycogen-bound water (ΔEBW₍gly₎) based on assumed release of either 1 g or 3 g H₂O per g glycogen. All masses are expressed in grams, where positive values indicate gains and negative values indicate losses.

Net change in body mass (ΔBM; post – pre, g; negative values indicate loss) was determined by measuring the athlete’s BM immediately before (cycling: 11:30 am; swim: 9:00 pm; roller-ski: 10:30 pm; trail run: 3:32 am) and after (cycling: 7:18 pm; swim: 9:48 pm; roller-ski: 2:03 am; trail run: 6:18 am) each stage using a calibrated digital scale (±0.100 g). When clothing was worn at either measurement, nude-equivalent mass was obtained by applying dry‑mass corrections based on recorded garment masses (cycling shorts 188 g; wetsuit 1000 g; swim shoes 138 g; swim briefs 65 g; socks 59 g; long‑sleeve 118 g; running shoes 449 g; cap 100 g). The wristwatch and heart‑rate strap were worn consistently across all measurements and were not adjusted.

Mass loss attributable to substrate oxidation (carbohydrate (CHO) and fat) during the cycling stage was quantified numerically integrating oxidation rates over time (trapezoidal method; Tai, 1994) to obtain total grams of CHO and fat oxidized. Instantaneous oxidation rates were derived from the athlete’s individualized linear relationships between VO₂, VCO₂, and external power, established during a graded cycling test (typical fit R² = 0.99). CHO and fat oxidation rates were then computed using standard stoichiometric equations assuming negligible protein oxidation (Jeukendrup & Wallis, 2005). The corresponding mass loss from substrate oxidation was taken as the mass of substrate oxidized (g). For swimming, roller skiing, and running, fixed fat oxidation rates of 0.5 g·min⁻¹ (swimming) and 1.1 g·min⁻¹ (roller skiing and running) were applied, based on data from top-amateur ultra-endurance triathletes (Maunder et al., 2018). Total CHO oxidation for each event was estimated by subtracting the energy derived from fat oxidation from total EE, and dividing by exercise duration. Energy equivalents of 9.75 kcal·g⁻¹ (fat) and 4.07 kcal·g⁻¹ (CHO) were applied. (Jeukendrup & Wallis, 2005).

Metabolic (cellular) water gain from substrate oxidation was assumed to be 0.600 g H₂O·g⁻¹ CHO oxidized and 1.085 g H₂O·g⁻¹ fat oxidized(Jeukendrup & Wallis, 2005).

Total glycogen utilization (g) was estimated by assuming that 80% of total CHO oxidation originated from muscle glycogen and 20% from plasma glucose. Water released during glycogen metabolism was estimated using two separate assumptions: (a) 1 g H₂O·g⁻¹ glycogen, and (b) 3 g H₂O·g⁻¹ glycogen, representing conservative and upper-bound estimates, respectively.

Total fluid intake (TFI) was defined as the sum of ad libitum water intake, all beverages (including sports drinks and gels), and moisture content of solid foods ingested during each event.

Urine output was estimated based on recorded voiding times, assuming a constant flow rate of 135 mL (≈135 g) per 5 s.

Detailed equations for estimating sweat secretion, ΔEBW, ΔEBWgly (1:1 and 3:1) are summarized in Supplementary Table S1.

**Supplementary Table S1**. Equations for sweat loss and effective body water (EBW) with and without glycogen‑associated water

| **Variable** | **Equation** | **Definition/References** |
| --- | --- | --- |
| **Body Mass and Effective Body Water Changes** | | |
| ΔEBW | (TFI) + (water generated during substrate oxidation) – (sweat secreted) – (urine excreted) | Does not include water bound with glycogen; Armstrong et al. (2015); King et al. (2008) |
| ΔEBWgly (1:1) | (TFI) + (water generated during substrate oxidation) + (1 g H2O released/1 g glycogen) – (sweat secreted) – (urine excreted) | Includes water bound with glycogen; Armstrong et al. (2015) |
| ΔEBWgly (3:1) | (TFI) + (water generated during substrate oxidation) + (3 g H2O released/1 g glycogen) – (sweat secreted) – (urine excreted) | Includes water bound with glycogen; Armstrong et al. (2015) |
| Sweat secreted | (ΔBM) + (ingested fluid mass) + (ingested solid food mass) + (water generated during substrate oxidation) – (mass loss due to substrate oxidation) – (urine excreted) | Armstrong et al. (2015) |
| Equation for low intensity exercise (40–50% VO₂max) | CHO oxidation (g·min⁻¹) = 4.344 × V̇CO₂ − 3.061× V̇O₂ | Jeukendrup and Wallis (2005) |
| Equation for moderate to high intensity (50–75% VO₂max) | CHO oxidation (g·min⁻¹) = 4.210 × V̇CO₂ − 2.962 × V̇O₂ | Jeukendrup and Wallis (2005) |
| Equation (all exercise intensities) | Fat oxidation (g·min⁻¹) = 1.695 × V̇O₂ − 1.701 × V̇CO₂ | Péronnet and Massicotte (1991) |

Abbreviations: ΔEBW, Net effective body water (excludes glycogen-bound water); ΔEBW (1:1) and ΔEBWgly (3:1), change in effective body water including 1 g or 3 g H₂O released per 1 g glycogen, respectively; ΔBM, Net change in body mass; TFI, total fluid intake; CHO, carbohydrate; V̇O₂, oxygen uptake (L·min⁻¹) V̇CO₂, carbon dioxide production (L·min⁻¹); VO₂max, maximal oxygen uptake; H₂O, water.

## **Pre- and Post-Event Oral Glucose Tolerance Test (OGTT)**

A standardized 120-min, 75-g oral glucose tolerance test (OGTT) was performed before and after the event following an overnight fast (≥9 h), with testing commencing at 08:00. Capillary blood glucose was measured every 15 min over 120 min. The athlete remained seated throughout all resting measurements.

**Glucose load and sampling schedule.** After a baseline capillary blood sample (0 min), the athlete ingested 300 mL of a 75 g glucose solution (dextrose monohydrate; Topstar 75 lemon, Esteriplas Ltd, Portugal). Capillary blood glucose was then measured (Biosen C-line, EKF diagnostics GmbH, Magdeburg, Germany) every 15 min across 120 min (0–120 min).

**Indirect calorimetry during OGTT.** To quantify substrate oxidation at rest, indirect calorimetry (Quark RMR/CPET; COSMED, Rome, Italy) was performed in 5-min bouts every 15 min across the OGTT (eight measurements total). For each bout, the mean of the final 60 s was used for all calculations. Respiratory exchange ratio (RER) was derived from concurrent VȮ₂ and VCȮ₂.

**Substrate oxidation.** Stoichiometric equations were applied to calculate carbohydrate (CHO) and fat oxidation rates from VȮ₂ and VCȮ₂ (L·min⁻¹), assuming negligible protein oxidation at rest (see Supplementary Table S2 for equations and references).

**Supplementary Table S2.**

| **Variable** | **Equation** | **References** |
| --- | --- | --- |
| Carbohydrate oxidation (g·min⁻¹) | CHO₍ox₎ = 4.585 × V̇CO₂ − 3.226 × V̇O₂ | Péronnet and Massicotte (1991) |
| Fat oxidation (g·min⁻¹) | Fat₍ox₎ = 1.695 × V̇O₂ − 1.701 × V̇CO₂ | Péronnet and Massicotte (1991) |

Abbreviations. CHO, carbohydrate; OGTT, oral glucose tolerance test; V̇O₂, oxygen uptake; V̇CO₂, carbon dioxide production.

**Metabolic flexibility (MetF).** MetF was operationalized as the change in RER from 0 to 60 min after glucose ingestion (δRER = RER60 min_{60\,min}60min​ – RER0 min_{0\,min}0min​).

**Glycemic response.** Capillary glucose responses were summarized as total AUC0–120_{0–120}0–120​ using the trapezoidal rule (computations performed in Excel; Tai, 1994).

# Supplementary Figures

**Suppl Figure S1.** Perceptions of gastrointestinal (GI) symptoms before (Pre) and after (Post) each event during the tetrathlon. Ratings were provided for overall GI comfort, abdominal pain, gas/bloating, stomach rumble, nausea, urge to urinate, and urge to empty bowel. For all GI symptoms except GI comfort, 0 = no symptoms and 20 = worst conceivable symptoms; for GI comfort, 0 = extremely uncomfortable and 20 = extremely comfortable.


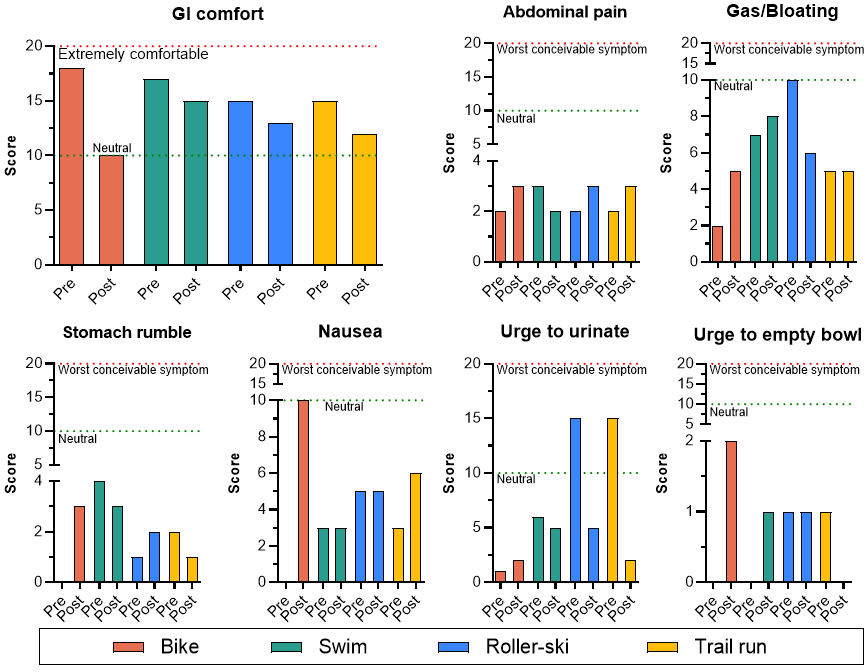


# References

Armstrong, L. E., Johnson, E. C., Ganio, M. S., Judelson, D. A., Vingren, J. L., Kupchak, B. R., Kunces, L. J., Muñoz, C. X., McKenzie, A. L., & Williamson, K. H. (2015). Effective body water and body mass changes during summer ultra-endurance road cycling. *J Sports Sci*, *33*(2), 125-135. <https://doi.org/10.1080/02640414.2014.932918>

Homestead, E. P., Peterman, J. E., Kane, L. A., Contini, E. J., & Byrnes, W. C. (2016). Estimating Energy Expenditure using Individualized, Power-Specific Gross Efficiencies. *Int J Sports Med*, *37*(14), 1129-1135. <https://doi.org/10.1055/s-0042-110655>

Jeukendrup, A. E., & Wallis, G. A. (2005). Measurement of substrate oxidation during exercise by means of gas exchange measurements. *Int J Sports Med*, *26 Suppl 1*, S28-37. <https://doi.org/10.1055/s-2004-830512>

King, R. F., Cooke, C., Carroll, S., & O'Hara, J. (2008). Estimating changes in hydration status from changes in body mass: considerations regarding metabolic water and glycogen storage. *J Sports Sci*, *26*(12), 1361-1363. <https://doi.org/10.1080/02640410802192768>

Le Nevé, B., Brazeilles, R., Derrien, M., Tap, J., Guyonnet, D., Ohman, L., Törnblom, H., & Simrén, M. (2016). Lactulose Challenge Determines Visceral Sensitivity and Severity of Symptoms in Patients With Irritable Bowel Syndrome. *Clin Gastroenterol Hepatol*, *14*(2), 226-233.e221-223. <https://doi.org/10.1016/j.cgh.2015.09.039>

Maunder, E., Kilding, A. E., & Plews, D. J. (2018). Substrate Metabolism During Ironman Triathlon: Different Horses on the Same Courses. *Sports Med*, *48*(10), 2219-2226. <https://doi.org/10.1007/s40279-018-0938-9>

Péronnet, F., & Massicotte, D. (1991). Table of nonprotein respiratory quotient: an update. *Can J Sport Sci*, *16*(1), 23-29.

Tai, M. M. (1994). A mathematical model for the determination of total area under glucose tolerance and other metabolic curves. *Diabetes Care*, *17*(2), 152-154. <https://doi.org/10.2337/diacare.17.2.152>
